# Understanding Family Tourism: A Perspective of Bibliometric Review

**DOI:** 10.3389/fpsyg.2022.937312

**Published:** 2022-07-04

**Authors:** Guanghui Qiao, Yating Cao, Qingwen Chen, Qiaoran Jia

**Affiliations:** ^1^School of Tourism and Urban-Rural Planning, Zheshang Research Institute, Academy of Zhejiang Culture Industry Innovation and Development, Zhejiang Gongshang University, Hangzhou, China; ^2^Zheshang Research Institute, Zhejiang Gongshang University, Hangzhou, China; ^3^School of International Tourism and Management, City University of Macau, Macao SAR, China

**Keywords:** family tourism, CiteSpace, seniors, disadvantaged families, bibliometric review

## Abstract

The study performed bibliometric visual analyses of family tourism research literature from 2008 to 2021, revealing the knowledge evolution process, research focuses, and future trends in this field. A total of 132 articles on family tourism were collated from the SSCI database of the Web of Sciences core collection and analyzed by CiteSpace. The results show that the number of research studies on family tourism has increased from 2008 to 2021, however, the overall base is small. Purdue University has the highest number of publications and citations. Inter-country cooperation occurs between the United States, China, the United Kingdom, and Australia. Recently, “motivation” and “benefit” have become hot topics in family tourism research, and “social tourism” has received widespread attention, revealing future research directions. Lehto and Wu are the core figures in the family tourism field, and their achievements have been highly cited and peer-recognized. This study focuses on family tourism research in different cultural situations, enriching the knowledge system of family tourism research, and encouraging future family tourism research focus more on seniors and disadvantaged families.

## Introduction

With the continuous growth of disposable family income and tourism promotion for family functions, the family tourism market has developed rapidly and has become the most important segment of the global tourism industry (Schänzel and Yeoman, 2015). For families, tourism has become a necessity rather than a luxury. It enables family members to spend unforgettable time together and create collective memories, thereby forming an effective connection (Lehto et al., 2009; Carr, 2011).

With the expansion of the family tourism market, tourism scholars have gradually paid attention to research on family tourism. The content of studies includes family tourism decision-making (Barlés-Arizón et al., 2013; Yang et al., 2020; Wang and Li, 2021), family tourism motivation (Kim and Lehto, 2013; Wang et al., 2018), family tourism benefit (Shaw et al., 2008; Lehto et al., 2009), and family tourism experience (Rhoden et al., 2016; Wu et al., 2019). From the perspective of research objects, some studies focus on the roles of couples in the family (Rojas-de-Gracia and Alarcón-Urbistondo, 2017). Others compare the perspectives of parents and children (Fu et al., 2014), and more studies have begun to focus on the voice of children in family tourism (Khoo-Lattimore, 2015; Rhoden et al., 2016). Previous studies on family tourism have mainly focused on western nuclear families. With the complexity of social structure, numerous changes have taken place in family structure. Many special types of families have emerged, such as families with disabled children (Kim and Lehto, 2013; Sedgley et al., 2017) and immigrant families (Yankholmes et al., 2021). In addition, in family tourism research, Chinese families influenced by traditional Confucian cultural values and one-child policy differ from western nuclear families (Wu and Wall, 2016a). Chinese family tourism reflects this view insofar as tourists pay more attention to children’s learning experiences (Wu and Wall, 2016b; Lehto et al., 2017) and the tourism preferences and needs of elderly parents (Wang et al., 2018). Therefore, there is a need to conduct a bibliometric study of family tourism research. Bibliometric studies identify the contribution to the knowledge and the development process of relevant fields by combing and reviewing the existing research literature, revealing the current research focuses and future research trends (Denyer and Tranfield, 2006).

Visualization tools can generate figures and tables to help in clarifying the complex relationship between a large numbers of research samples. Visualization is crucial in the field of knowledge. It can help researchers to quickly understand the development of relevant research fields (Speel et al., 1999). This method has not been fully used in tourism research. When used effectively, it can explore the network structure of different tourism environments (Scott et al., 2008).

This study aims to conduct a detailed search of family tourism research literature in the SSCI database of the Web of Sciences core collection from 2008 to 2021 and to analyze with CiteSpace, a popular knowledge domain visualization tool (Chen et al., 2010). This will provide a better direction for the follow-up research and improve the knowledge structure of international family tourism. This study also attempts to highlight seniors, a neglected voice in family tourism. The extension of the average life expectancy and of social development make the intergenerational relationship between grandparents and grandchildren increasingly important in family life, and the number of “multi-generational holidays” and “grandtravel” groups is increasing (Gram et al., 2019). This situation is particularly prominent in family tourism with Chinese cultural context, in which most adult children maintain close ties with their parents, grandparents help to take care of their grandchildren, and adult children provide care and spiritual comfort for their parents (Gruijters, 2017; Wang et al., 2018). Older people need a sense of security, family affection, and belonging. Adult children take filial piety as a natural responsibility. Taking parents on trips has become a way of showing their gratitude and filial piety (Wang et al., 2018). There is also growing research attention on disadvantaged families, such as families with disabled family members. Continuous improvements in social welfare offer disadvantaged family groups more opportunities to participate in tourism activities, thereby forming a potentially important niche market.

## Methodology

### Data Collection

This study uses the SSCI database of the Web of Sciences core collection as the data source. The first search is by topic, (TS) = “family tourism” or “family travel,” in which “topic” covers the title, summary, and keywords of an article. Then, there are 10 searches for “family,” “parents,” “children,” “grandparents,” and “couples,” each combined with “tourism” and “travel.” The searches yielded 3,759 records (on November 20, 2021). Through reading the titles, keywords, and abstracts of each of these outputs, out-of-scope literature (e.g., transport and school travel, *n* = 1,343, family tourism enterprises, *n* = 272, medical and birth tourism, *n* = 654, etc.) was eliminated. Finally, a sample of 132 articles spanning 2008–2021 was retained.

### Data Analysis

CiteSpace is a bibliometric visualization software package developed with Java language, which is used as a tool for scientific and technical text mining and analysis. It can reveal the knowledge evolution process of a specific field by drawing a series of visualization maps. CiteSpace helps to analyze pioneering and iconic literature in a research field, hot topics in the field, and the evolution of research frontiers.

CiteSpace calculates several indicators, including betweenness centrality, modularity (Q), and mean silhouette (S). Research literature with betweenness centrality greater than 0.1 is located at the center of the network, connecting different knowledge subfields (Chen et al., 2014). CiteSpace provides two indicators, modularity (Q) and silhouette (S), to judge the mapping effect. Q is generally within the range of 0–1. *Q* > 0.3 means that the clustering structure is significant, and clustering with S above 0.5 is usually considered reasonable (Chen et al., 2014). Modularity and contour values should be considered simultaneously to ensure a reasonable explanation for the clustering characteristics of networks in CiteSpace (Chen et al., 2010). CiteSpace provides researchers with various bibliometric networks, including cooperation, keyword co-occurrence, and co-citation networks (Li and Chen, 2017). In tandem with the network structure and content, CiteSpace’s burst detection function helps us find special points and identify keywords or articles repeatedly mentioned by scholars in a certain period (Chen, 2006).

These maps can show the development status and changes in scientific structure, and they are used for frontier analysis, field analysis, and scientific research evaluation. This study adopts the following analysis methods: co-citation analysis of the research literature, journals, and authors; network of coauthors’ institutions and countries; and co-occurrence analysis of keywords.

## Results and Discussion

### Network of Journals

**FIGURE 1:**
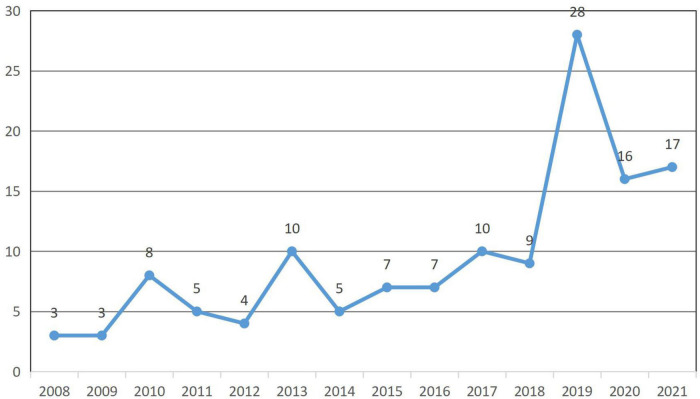
Year-wise publication from Web of Sciences.

Figure 1 shows that the average annual publication volume on family tourism from 2008 to 2012 was small. The number of studies increased significantly from 2012 to 2013, reaching a small peak, falling back in 2014 to the 2012 level. Since then, the number of studies has grown slowly, with a sharp increase from 2018 to 2019, reaching 28. From 2019 to 2021, the level was relatively high at 15 articles. It seems that more and more scholars pay attention to family tourism, but the overall number of studies remains small, and there is extensive untapped scale and scope for future research.

The number of citations of journals reflect the influence of journals (Garfield, 1972). Therefore, the journals were ranked based on careful consideration of the total number of citations and articles (Table 1), and the top 10 journals were analyzed. *Annals of Tourism Research* published nine articles, with 72 citations and an average citation score of 8.00. Although only one article each was published by *Leisure Science* and the *Journal of Hospitality Marketing and Management*, the number of citations was high. *Tourism Review* currently has one relevant article, indicating that the journal has begun to pay attention to research on family tourism. Of the analyzed studies, 45.1% were published in the top 10 journals, accounting for 84.9% of the total citations of all the analyzed literature, indicating that these journals are influential in family tourism research.

**TABLE 1:** Top Journals in family tourism research.

	Total		Citation per	Journal
			
Source title	Publications	Citations	Publications	Impact factor
Annals of Tourism Research	9	72	8.00	9.011
Tourism Management	14	53	3.79	10.967
Journal of Travel and Tourism Marketing	12	48	4.00	7.564
Current Issues in Tourism	5	21	4.20	7.430
Journal of Travel Research	5	20	4.00	10.982
Leisure Sciences	1	19	19.00	2.750
Journal of Hospitality Marketing and Management	1	15	15.00	7.022
Tourism Management Perspectives	8	11	1.38	6.586
Journal of Hospitality and Tourism Research	3	8	2.67	5.161
Scandinavian Journal of Hospitality and Tourism	2	8	4.00	4.392
Total	60	275		
(%)	45.1	84.9		

*Source: Self complied.*

**FIGURE 2:**
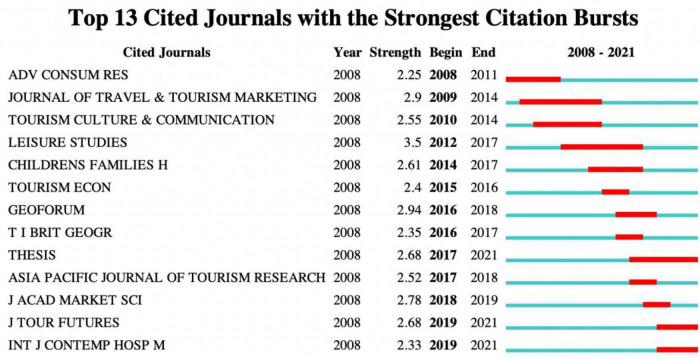
Citation bursts of the top 13 journals.

The index of emergent citations shows the active degree of journals. Figure 2 shows the most frequently cited journals in the same year. At present, *Leisure Studies* has the most citations, followed by *Geoforum*. The longest citation bursts were for the *Journal of Travel and Tourism Marketing* and *Leisure Studies*, which lasted for 6 years. In recent years, the citation frequency of *Thesis*, *Journal of Tourism Futures*, and *International Journal of Contemporary Hospitality Management* has become more significant, showing that more journals are involved in family tourism research.

### Collaborations Between Institutions

From Table 2, the co-operation network among institutions has a high modularity and mean silhouette, and the network graph is of good quality. As time goes by, there are more nodes and links between them in the network, but the overall density value is low, which indicates that institutions increasingly have paid attention to family tourism as a research field, however, a central institution has not been formed to guide other institutions, and the collaboration between institutions in family tourism is scattered.

**TABLE 2:** CiteSpace metrics by co-operation institutions network.

Network	Node type by year	Modularity	Nodes	Links	Density	#Clusters	Mean silhouette
Co-operation institutions	2008–2015	0.9423	71	47	0.0189	35	0.8028
	2016–2021	0.8892	105	105	0.0192	44	0.7859

*Source: Self complied.*

As Table 3 shows, Purdue University has the most achievements in family tourism research, with the highest number of papers and citations. It is also the institution that publishes the most papers in the first unit, followed by Zhejiang University. Although the number of papers published by Texas A&M University and Griffith University is not large, the average citation scores rank toward the top, indicating that the research results of these institutions are of high quality. Overall, the United States has the largest number of institutions, followed by China and the United Kingdom.

**TABLE 3:** Top institutions in family tourism research.

Rank	Institutions	Publications	Citations	TC/TP	First authors	Countries
1	Purdue University	16	140	8.75	8	United States
2	Zhejiang University	11	31	2.82	6	China
3	Hong Kong Polytech University	6	8	1.33	2	China
4	Texas A&M University	4	34	8.50	2	United States
5	Griffith University	4	30	7.50	3	Australia
6	University Nottingham	4	17	4.25	1	United Kingdom
7	University Queensland	4	0	0.00	1	Australia
8	University Cent Florida	3	10	3.33	2	United States
9	University Waterloo	3	9	3.00	1	Canada
10	Coll Charleston	3	9	3.00	1	United States
11	University Malaga	3	6	2.00	3	Span
12	Sheffield Hallam University	3	5	1.67	3	United Kingdom
13	University Surrey	3	4	1.33	1	United Kingdom
14	Oklahoma State University	3	4	1.33	0	United States
15	Jinwen University Sci and Technol	3	4	1.33	1	China
16	Penn State University	3	4	1.33	1	United States
17	Abdul Wali Khan University	3	3	1.00	1	Pakistan
18	College Holy Cross	3	1	0.33	3	United States

*Source: Author made.*

### Collaborations Between Countries

This study analyzes the author’s national network to explore whether family tourism research forms a stable cooperative relationship between different countries or regions. From Table 4, there are more nodes in the network and the connections between them. Over time, researchers from more countries participate in family tourism research, and the international collaboration between them is more complex. The density of the network shows an upward trend, and the number of clusters have also decreased from 15 to 10, indicating that the countries network structure is more concentrated.

**TABLE 4:** CiteSpace metrics by co-operation countries network.

Network	Node type by year	Modularity	Nodes	Links	Density	#Clusters	Mean silhouette
Co-operation countries	2008–2015	0.6529	24	13	0.0471	15	0.4607
	2016–2021	0.657	34	55	0.098	10	0.8507

*Source: Self complied.*

**FIGURE 3:**
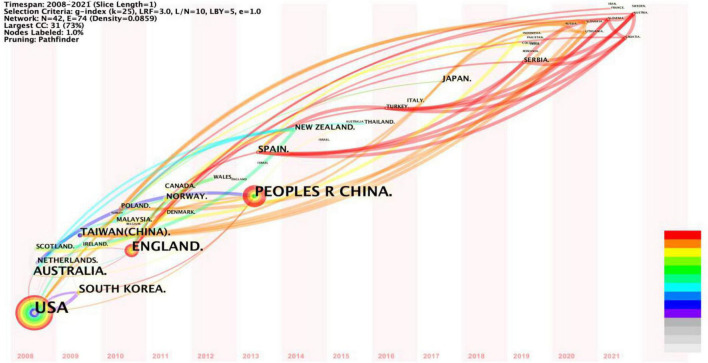
A time-zone view of the country/region collaboration network.

The time-zone view of the country/region collaboration network (Figure 3) shows that the United States, China, the United Kingdom, and Australia have laid the foundation for cooperation with other countries and regions. Emerging cooperation networks include the United States and France; China and South Korea; Japan, Poland, Russia, and Spain; Turkey, and Serbia, illustrating the increasing frequency of cooperation among some emerging market countries. However, in general, international collaboration in family tourism research needs to be improved, especially between European and Asian countries, so as to facilitate differentiated research within different cultural backgrounds.

### Research Themes

The co-occurrence analysis of keywords and keyword expansion allows us to see the knowledge structure of the subject (Ding et al., 2001). From 2008 to 2021, there were 225 keywords and keyword expansions, and 20% of them were used more than three times. This shows that the area where family tourism research is concentrated is relatively small.

**FIGURE 4:**
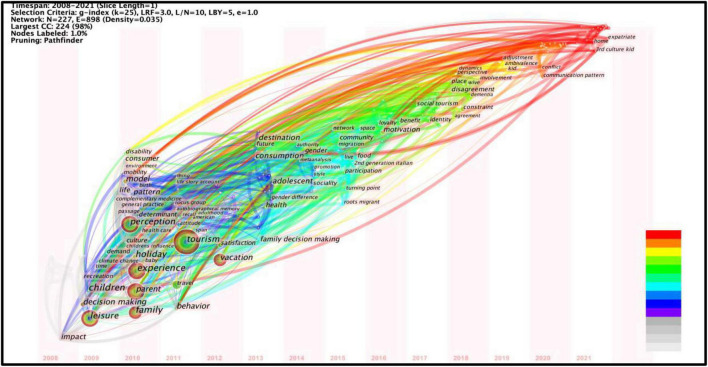
A time-zone view of keywords with high frequencies from 2008–2021.

Figure 4 shows that the keyword with the highest frequency is “tourism,” followed by “leisure,” “parent,” “perception,” “experience,” “family,” and “children.” From the time-zone view, we can see that the research topic has changed over time. From 2008 to 2012, the research topics covered family travel decision-making, participation, and experience, mainly from the perspectives of parents and children. Family leisure became the focus, and tourism became an important part of leisure activities. Since then, new research topics have emerged, such as family tourism destinations. Due to different motivations of family members, studies on family tourism types have gradually diversified, as in adventure tourism, heritage tourism, and ecological tourism, which, respectively, involve 6, 3 and 4 sample literatures. Among them, the main research directions of family adventure tourism are motivation and the impact of adventure on hedonic and well-being; heritage tourism mainly involves children’s experiential learning experience; ecological tourism mainly involves children’s nature conservation. This development is related to parents’ expectation that tourism will have educational significance for children. Tourism is considered to be the best education (Yang and Lau, 2019). As an informal learning method, tourism is more active, interactive and experiential (Shaw and Dawson, 2001). Therefore, learning experience has become one of the important motivations of family tourism.

In the past 5 years, “motivation” and “benefit” have gradually become research hotspots. Academia began to pay attention to the travel motives of disadvantaged family groups and the broader significance of family travel. It is worth noting that “social tourism” has become a new keyword in recent years, 17 sample literatures were involved, and the research objects included families with autistic children, low-income families, and families with the visually impaired, etc. As an emerging form of tourism and social policy, social tourism targets low-income families, enabling them to participate in tourism activities (Minnaert et al., 2011). This is consistent with the research on families of disabled children mentioned above. Disadvantaged families have been highlighted and have gradually been included in family tourism research.

**FIGURE 5:**
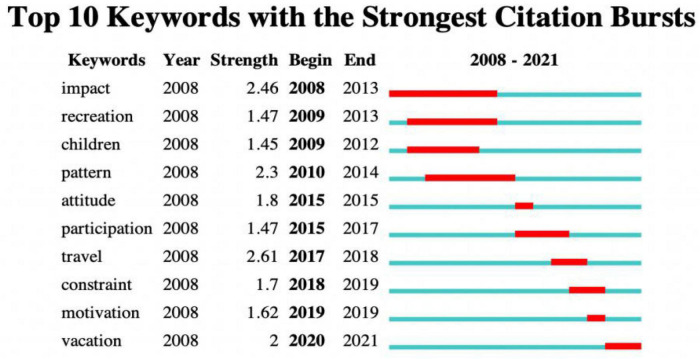
Top 10 keywords with the strongest citation bursts.

Figure 5 shows ten burst keywords that appeared from 2008 to 2021. This burst of keywords indicates an emerging trend (Chen et al., 2014). Since 2008, “impact” has become a burst keyword. This has continued up to 2013, indicating that family tourism has been of broad interest. During this time, “recreation,” “children,” and “pattern” have been mentioned frequently. Family tourism studies are focusing on children’s voices, and the influence of children’s tourism is increasingly influential. The focus on children also mainly around tourism decision, experience, benefit, etc. Relevant studies have proved that children have an increasing impact on family tourism decisions (Kozak, 2010). They do not passively obey the decisions made by their parents and play an important role in tourism product purchase and on-site decision-making (Blichfeldt et al., 2011). In addition, family tourism pays more attention to children’s experience (Rhoden et al., 2016), especially the pursuit of learning experience. And in the recent study of family tourism value, it has become a trend to pay attention to the eudaimonic wellbeing that breaks through challenges and realizes self-development, and the role of intergenerational tourism in promoting personal and intergenerational wellbeing. Subsequently, “attitude” and “participation” became burst keywords. “Travel” and “constraint” were often mentioned. Among them, the vacation tourism constraints of aging parents and families with disabilities have been paid frequently attention (Kong and Loi, 2017; Heimtun, 2019). In recent years (2019–2021), “motivation” and “vacation” have become breakout themes.

### Co-citation Analysis by Thematic Clusters

**FIGURE 6:**
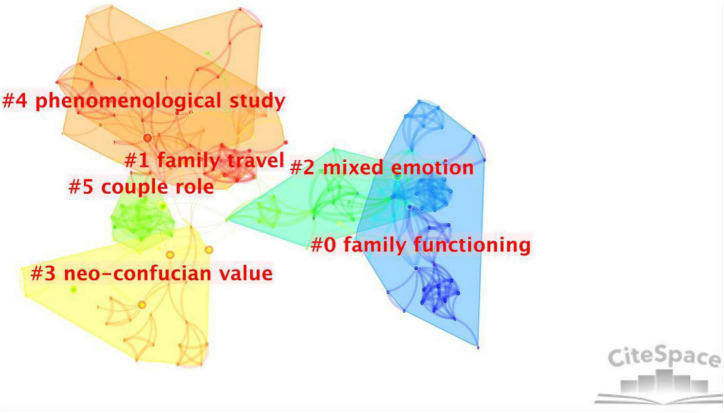
Cluster analysis of the reference co-citation network.

Co-citation clustering reflects the core themes and knowledge foundation of family tourism research. Figure 6 is a reference clustering diagram constructed using CiteSpace. In our study, six major clusters were identified and featured according to the title of the articles. The modularity (Q) of co-citation clusters in this study is 0.8622, and the mean silhouette (S) is 0.9275, which shows a significant visualization effect.

The most cited papers provide a historical perspective on and reveal the recognition of scientific progress (Chen, 2006). The more obvious the label is in front of the cluster block, the more articles are cited. There are 42 articles in the “#0 family functioning” cluster, and one article with a high citation level (Lehto et al., 2009) discusses the unique interaction among family vacation tourism, family cohesion, and family communication, showing that family vacations play a positive role in promoting family relations, communication, and unity. A study (Shaw et al., 2008) based on semi-structured interviews with family members in Ontario, Canada, discussed the cultural significance and experience of school-age children’s family vacations and proposed that the important aspect in family tourism is the creation of a long-term memory, which will strengthen family cohesion and establish and support positive family awareness. It can be seen that family tourism research from the functional perspective has laid a sound research foundation for family relations and children’s informal learning. There are 41 articles in the “#1 family travel” cluster, among which the two articles with the highest citation rates are from Wu and Wall (2016a) and Wu et al. (2019). In addition, an article on the family tourism motivation of Chinese adult children and parents (Wang et al., 2018) was completed by Wu and his colleagues. It can be seen that Wu holds a core position in this field, and that research on family tourism in the Chinese context has attracted extensive attention in the academic community. The articles in the “#2 mixed emotion” cluster mainly include the motivation, activities, and obstacles of families of disabled individuals. “#3 neo-Confucian values” has a distinct cultural background, and the research objects in the article are mainly Asian families. The articles contained in “#4 phenomenological study” mainly discuss the accommodation constraints and needs of European parents with children on vacation based on qualitative research and use innovative programming to measure the role of children in parents’ tourism decision-making. The articles in the “#5 couple role” cluster mainly discuss the role of the couple in tourism decisions and indicate that women are increasingly influencing family travel decisions.

### Articles With Citation Bursts

**FIGURE 7:**
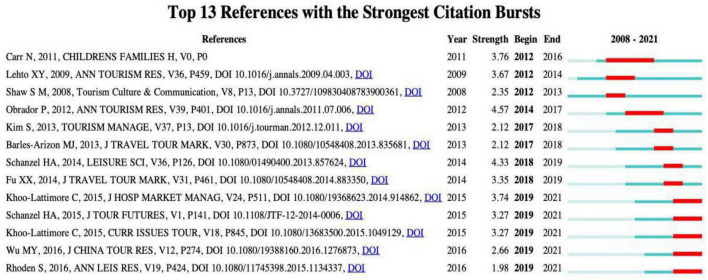
Top references with the strongest citations bursts.

**FIGURE 8:**
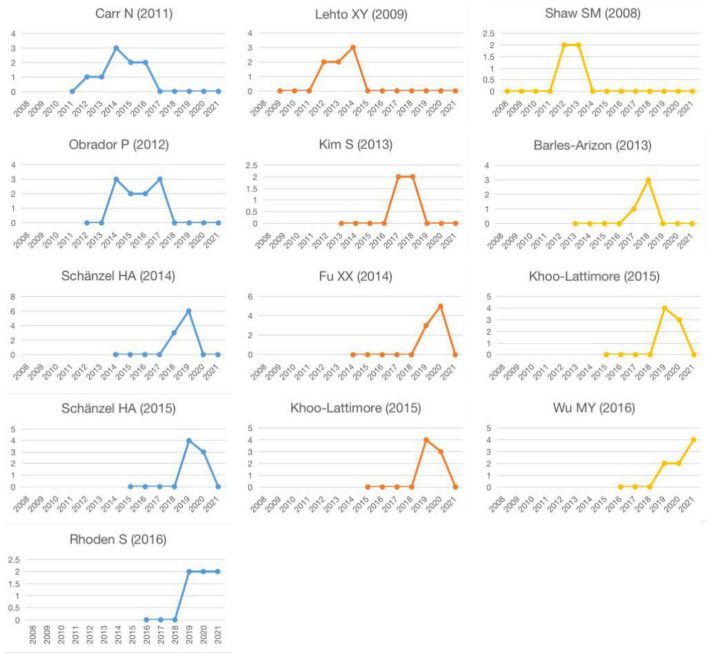
The annual citation count for top 13 references from publication year to 2021.

The burst of a cited paper reflects the dynamic evolution of research hotspots (Chen et al., 2014). Figure 7 shows the 13 most frequently cited studies in the same year, and Figure 8 shows the annual citation count for these top 13 cited reference from the publication to 2021. The burst of citations began in 2012, and the strongest outbreaks were led by Shaw et al. (2008); Lehto et al. (2009), and Carr (2011). Carr (2011) offered important opinions on the experience of family tourism, which guided follow-up research scholars and tourism managers. Shaw et al. (2008) and Lehto et al. (2009) mainly discussed the significance of family tourism in family unity, cohesion, and communication. The strongest burst in 2014 was a theoretical article by Obrador (2012), which focused on the position of the family in tourism research.

During the 2 years between 2017 and 2019, there was a continuous wave of citations about family tourism. The most prominent citations in 2017 were related to Barlés-Arizón et al. (2013) and Kim and Lehto (2013). The first one focused on the motivation and travel activities of families with disabled children. The second one analyzed the impact on family tourism decisions in couples on the woman side. The most flame-up research in 2018 came from Schänzel and Smith (2014). The article provided new insights into family tourism functions according to the multidisciplinary of group dynamics. In a full-family approach to 10 families in New Zealand, Fu et al. (2014) analyzed the impact of family tourism by comparing generational differences between parents and children. Five articles were especially noted in 2019. Two articles, published by Khoo-Lattimore in 2015 (Khoo-Lattimore, 2015; Khoo-Lattimore et al., 2015), emphasized the role of children in family tourism. Rhoden et al. (2016) also focused on the travel experience of children. Schänzel and Yeoman (2015) proposed the significance of ten changing trends for family tourism. Wu and Wall (2016a) reviewed the literature on family tourism in the Chinese cultural contest. It is noteworthy that three of the 13 articles are from Lehto and her colleagues. Lehto’s achievements in the field of family tourism are outstanding and have been widely recognized by peers.

## Conclusion

The family unit is the center of social activities. The most intimate and most important emotional bonds are formed with individuals’ children and families. Family tourism promotes the family system development and the harmony of society (Yeoman, 2008; Schänzel and Smith, 2014). Family tourism plays a vital role in the tourism market.

CiteSpace is a visualizing knowledge graph analysis software package, with more advantages than other bibliometric analysis tools. First, by measuring and visualizing the network of different nodes, the nuances of knowledge can be better revealed (Chen, 2006). Second, CiteSpace can decompose a variety of networks into automatically labeled clusters with terms from citing articles, enabling researchers to identify turning and pivotal points of knowledge domains. Third, the scientific cooperation network can be visualized. More collaborative connections indicate a very high rate of collaboration between research groups. Finally, the burst detection and co-occurrence analysis of high frequency and high betweenness centrality keywords can be used to identify research hotspots and trends. This research gives readers a clear picture of family tourism research topics and their evolution patterns in the past 14 years, finds research hotspots, and predicts trends by conducting collaboration network analysis, co-occurrence analysis, and co-citation analysis.

Through collaboration network analysis, the study provides an understanding and interpretation of the most cited authors and the active citation journals, including identifying the institutions and countries at the forefront of family tourism research. Through the co-occurrence analysis of keywords, we found that “motivation” and “benefit” had been popular keywords in the past 5 years. Related articles put significant attention on children, and some new viewpoints have been proposed, such as the travel motivation of senior parents and of families with disabled children. The co-citation analysis of the literature shows the dynamic evolution of family tourism research hotspots and helps readers to quickly interpret the basic and outstanding studies.

Family tourism research is affected by different cultural contests, social conditions, and economic development. Therefore, family tourism research scenarios need to be diversified to enrich their knowledge systems. The results of this study encourage future research to pay attention to the voices of elderly family members and the influence of family tourism on the physical and mental health, subjective well-being, and self-value realization of elderly family members. This research aims to fill the gap in the “parents-oriented” travel motivation study and give support to the promotion of active aging. In practice, this study offers tourism managers the ability to effectively understand current and future family tourism market needs and restrictions in order to propose more targeted products.

This study’s limitations are as follows. First, due to the constraints of CiteSpace, the sample literatures are limited to the SSCI from the WoS Core Collection, and the authors only selected articles and reviews in the collections, excluding other published works and there are some tourism journals and related articles missing. In order to alleviate this limitation, we discussed highly cited literatures in co-citation analysis. Besides, the language limitations of the research team confine this study to journals published in English. However, many literature on family tourism have been published in Korean, Chinese, Japanese, and other languages.

In future studies, it is necessary to further study family tourism in different family types, and pay attention to the travel preferences and needs of different family members, especially the seniors. In order to promote active aging, it is necessary to discuss the benefits of family tourism from the perspective of the seniors and help them achieve intergenerational happiness.

## Author Contributions

GQ: conceptualization, validation, formal analysis, investigation, writing – original draft preparation, and funding acquisition. YC: writing – original draft preparation, methodology, and data curation. QC: conceptualization, methodology, and data curation. QJ: data curation and review and editing. All authors contributed to the article and approved the submitted version.

## Conflict of Interest

The authors declare that the research was conducted in the absence of any commercial or financial relationships that could be construed as a potential conflict of interest.

## Publisher’s Note

All claims expressed in this article are solely those of the authors and do not necessarily represent those of their affiliated organizations, or those of the publisher, the editors and the reviewers. Any product that may be evaluated in this article, or claim that may be made by its manufacturer, is not guaranteed or endorsed by the publisher.

## References

[B1] Barlés-Arizón M. J. Fraj-Andrés E. Martínez-Salinas E. (2013). Family vacation decision making: the role of woman. *J. Travel Tour. Mark.* 30 873–890. 10.1080/10548408.2013.835681

[B2] Blichfeldt B. S. Pedersen B. M. Johansen A. Hansen L. (2011). Tweens on holidays in-situ decision-making from children’s perspective. *Scand. J. Hosp. Tour.* 11 135–149.

[B3] Carr N. (2011). *Children’s and Families’ Holiday Experience.* London, UK: Routledge. 10.4324/9780203832615

[B4] Chen C. (2006). Citespace II: detecting and visualizing emerging trends and transient patterns in scientific literature. *J. Am. Soc. Inf. Sci. Technol.* 57 359–397. 10.1002/asi.20317

[B5] Chen C. Dubin R. Kim M. C. (2014). Emerging trends and new developments in regenerative medicine: a scientometric update (2000–2014). *Expert Opin. Biol. Ther.* 14 1295–1317. 10.1517/14712598.2014.920813 25077605

[B6] Chen C. Ibekwe-SanJuan F. Hou J. (2010). The structure and dynamics of co-citation clusters: a multiple-perspective co-citation analysis. *J. Am. Soc. Inf. Sci. Technol.* 61 1386–1409. 10.1002/asi.21309

[B7] Denyer D. Tranfield D. (2006). Using qualitative research synthesis to build an actionable knowledge base. *Manag. Decis.* 44 213–227. 10.1136/bmjqs-2014-003642 26306609 PMC4680127

[B8] Ding Y. Chowdhury G. G. Foo S. (2001). Bibliometric cartography of information retrieval research by using co-word analysis. *Inf. Process. Manag.* 37 817–842. 10.1016/S0306-4573(00)00051-0

[B9] Fu X. Lehto X. Park O. (2014). What does vacation do to our family? Contrasting the perspectives of parents and children. *J. Travel Tour. Mark.* 31 461–475. 10.1177/019251387008001002 12281044

[B10] Garfield E. (1972). Citation analysis as a tool in journal evaluation. *Science* 178 471–479. 10.1126/science.178.4060.471 5079701

[B11] Gram M. O’Donohoe S. Schänzel H. Marchant C. Kastarinen A. (2019). Fun time, finite time: temporal and emotional dimensions of grandtravel experiences. *Ann. Tour. Res.* 79:102769. 10.1016/j.annals.2019.102769

[B12] Gruijters R. J. (2017). Intergenerational contact in Chinese families: structural and cultural explanations. *J. Marriage Fam.* 79 758–768.

[B13] Heimtun B. (2019). Holidays with aging parents: pleasures, duties and constraints. *Ann. Tour. Res.* 76 129–139.

[B14] Khoo-Lattimore C. (2015). Kids on board: methodological challenges, concerns and clarifications when including young children’s voices in tourism research. *Curr. Issues Tour.* 18 845–858. 10.1080/13683500.2015.1049129

[B15] Khoo-Lattimore C. Prayag G. Cheah B. L. (2015). Kids on board: exploring the choice process and vacation needs of Asian parents with young children in resort hotels. *J. Hosp. Mark. Manag.* 24 511–531. 10.1080/19368623.2014.914862

[B16] Kim S. Lehto X. Y. (2013). Travel by families with children possessing disabilities: motives and activities. *Tour. Manag.* 37 13–24. 10.1016/j.tourman.2012.12.011

[B17] Kong W. H. Loi K. I. (2017). The barriers to holiday-taking for visually impaired tourists and their families. *J. Hosp. Tour. Manage.* 32 99–107.

[B18] Kozak M. (2010). Holiday taking decisions—the role of spouses. *Tour. Manag.* 31 489–494. 10.1016/j.tourman.2010.01.014

[B19] Lehto X. Y. Choi S. Lin Y. C. MacDermid S. M. (2009). Vacation and family functioning. *Ann. Tour. Res.* 36 459–479. 10.1016/j.annals.2009.04.003

[B20] Lehto X. Y. Fu X. Li H. Zhou L. (2017). Vacation benefits and activities”. *J. Hosp. Tour. Res.* 41 301–328. 10.1177/1096348013515921

[B21] Li J. Chen C. M. (2017). *CiteSpace: Text Mining and Visualization in Scientific Literature*, 2nd Edn. Beijing: Capital University of Economics and Business Press.

[B22] Minnaert L. Maitland R. Miller G. (2011). What is social tourism. *Curr. Issues Tour.* 14 403–415. 10.1080/13683500.2011.568051

[B23] Obrador P. (2012). The place of the family in tourism research: domesticity and thick sociality by the pool. *Ann. Tour. Res.* 39 401–420. 10.1016/j.annals.2011.07.006

[B24] Rhoden S. Hunter-Jones P. Miller A. (2016). Tourism experiences through the eyes of a child. *Ann. Leis. Res.* 19 424–443. 10.1080/11745398.2015.1134337

[B25] Rojas-de-Gracia M.-M. Alarcón-Urbistondo P. (2017). Couple roles in subdecisions on family vacations. *Cornell Hosp. Q.* 59 160–173. 10.1016/j.dib.2019.104233 31453280 PMC6702380

[B26] Schänzel H. Yeoman I. (2015). Trends in family tourism. *J. Tour. Futures* 1 141–147. 10.1108/JTF-12-2014-0006

[B27] Schänzel H. A. Smith K. A. (2014). The socialization of families away from home: group dynamics and family functioning on holiday. *Leis. Sci.* 36 126–143. 10.1080/01490400.2013.857624

[B28] Scott M. Baggio R. Cooper C. (2008). *Network Analysis and Tourism: from Theory to Practice.* Buffalo, NY: Channel View Publications. 10.21832/9781845410896

[B29] Sedgley D. Pritchard A. Morgan N. Hanna P. (2017). Tourism and autism: journeys of mixed emotions. *Ann. Tour. Res.* 66 14–25. 10.1016/j.annals.2017.05.009

[B30] Shaw S. M. Dawson D. (2001). Purposive leisure: examining parental discourses on family activities. *Leis. Sci.* 23 217–231.

[B31] Shaw S. M. Havitz M. E. Delemere F. M. (2008). I decided to invest in my kids’ memories: family vacations, memories, and the social construction of the family. *Tour. Cult. Commun.* 8 13–26. 10.3727/109830408783900361 30089248

[B32] Speel P.-H. Shadbolt N. de Vries W. Van Dam P. H. O’Hara K. (1999). “Knowledge mapping for industrial purposes,” in *Proceedings of the 12th Workshop on Knowledge Acquisition Modeling and Management*, Banff AB.

[B33] Wang W. Yi L. Wu M. Y. Pear Ce P. L. Huang S. S. (2018). Examining Chinese adult children’s motivations for traveling with their parents. *Tour. Manag.* 69 422–433. 10.1016/j.tourman.2018.06.024

[B34] Wang Y. Li M. (2021). Family identity bundles and holiday decision making. *J. Travel Res.* 60 486–502. 10.1177/0047287520930091

[B35] Wu M. Y. Wall G. (2016a). Chinese research on family tourism: review and research implications. *J. China Tour. Res.* 12 274–290. 10.1080/19388160.2016.1276873

[B36] Wu M. Y. Wall G. (2016b). Visiting heritage museums with children: chinese parents’ motivations. *J. Herit. Tour.* 12 36–51. 10.1080/1743873X.2016.1201085

[B37] Wu M. Y. Wall G. Zu Y. Ying T. (2019). Chinese children’s family tourism experiences. *Tour. Manag. Perspect.* 29 166–175. 10.1016/j.tmp.2018.11.003

[B38] Yang F. X. Lau M. C. (2019). Experiential learning for children at world heritage sites: the joint moderating effect of brand awareness and generation of Chinese family travelers. *Tour. Manage.* 72 1–11.

[B39] Yang M. Khoo-Lattimore C. Yang E. (2020). Three generations on a holiday: exploring the influence of neo-Confucian values on Korean multigenerational family vacation decision making. *Tour. Manag.* 78:104076. 10.1016/j.tourman.2020.104076

[B40] Yankholmes A. McKercher B. Williams N. L. (2021). A latent class approach to examining migrant family travel behavior. *Tour. Manag.* 87:104387. 10.1016/j.tourman.2021.104387

[B41] Yeoman I. (2008). *Tomorrow’ s Tourist: Scenarios and Trends.* Oxford, UK: Elsevier. 10.1016/S1572-560X(08)00434-4

